# The long noncoding RNA TUG1 regulates blood-tumor barrier permeability by targeting miR-144

**DOI:** 10.18632/oncotarget.4331

**Published:** 2015-06-02

**Authors:** Heng Cai, Yixue Xue, Ping Wang, Zhenhua Wang, Zhen Li, Yi Hu, Zhiqing Li, Xiuli Shang, Yunhui Liu

**Affiliations:** ^1^ Department of Neurosurgery, Shengjing Hospital of China Medical University, Shenyang, People's Republic of China; ^2^ Department of Neurobiology, College of Basic Medicine, China Medical University, Shenyang, People's Republic of China; ^3^ Institute of Pathology and Pathophysiology, China Medical University, Shenyang, People's Republic of China; ^4^ Department of Physiology, College of Basic Medicine, China Medical University, Shenyang, People's Republic of China; ^5^ Department of Neurology, The First Affiliated Hospital of China Medical University, Shenyang, People's Republic of China

**Keywords:** glioma, TUG1, microRNA-144, blood-tumor barrier, HSF2

## Abstract

Blood-tumor barrier (BTB) limits the delivery of chemotherapeutic agent to brain tumor tissues. Long non-coding RNAs (lncRNAs) have been shown to play critical regulatory roles in various biologic processes of tumors. However, the role of lncRNAs in BTB permeability is unclear. LncRNA TUG1 (taurine upregulated gene 1) was highly expressed in glioma vascular endothelial cells from glioma tissues. It also upregulated in glioma co-cultured endothelial cells (GEC) from BTB model in vitro. Knockdown of TUG1 increased BTB permeability, and meanwhile down-regulated the expression of the tight junction proteins ZO-1, occludin, and claudin-5. Both bioinformatics and luciferase reporter assays demonstrated that TUG1 influenced BTB permeability via binding to miR-144. Furthermore, Knockdown of TUG1 also down-regulated Heat shock transcription factor 2 (HSF2), a transcription factor of the heat shock transcription factor family, which was defined as a direct and functional downstream target of miR-144. HSF2 up-regulated the promoter activities and interacted with the promoters of ZO-1, occludin, and claudin-5 in GECs. In conclusion, our results indicate that knockdown of TUG1 increased BTB permeability via binding to miR-144 and then reducing EC tight junction protein expression by targeting HSF2. Thus, TUG1 may represent a useful future therapeutic target for enhancing BTB permeability.

## INTRODUCTION

Glioblastoma multiforme (GBM) is one of the most lethal primary malignant brain tumors. The existence of blood-tumor barrier (BTB) contributes to the failure of conventional chemotherapy by restricting sufficient drug molecules delivery to tumor tissues [1-3]. Therefore, selectively open the BTB without effecting normal blood-brain barrier (BBB) is considered promising future therapeutic strategies for effective chemotherapy of glioma.

Drugs cross the BTB by two pathways: paracellular or transcellular [4]. The transcellular pathway is the main route of absorption for chemotherapy drug molecules [5, 6]. The paracellular pathway is composed of the tight junction complex (TJs) including zonula occludens (ZOs), transmembrane proteins of occludin and claudins, et al [7].

In recent years, non-coding RNAs (ncRNAs) including long non-coding RNAs (lncRNAs) and MicroRNAs (miRNAs), have recently gained significant attention in tumor malignant processes such as carcinogenesis, metastasis and angiogenesis [8-10]. LncRNAs are defined as a class of more than 200 nucleotides non-coding RNAs that regulate various biologic processes of tumor. Some carcinogenic lncRNAs, including H19, CASC2 and HOTAIR, are found to be dysregulated and considered as promising future therapeutic strategies for gliomas [11-13]. MicroRNAs, new class of short non-coding RNAs, which negatively regulate gene expression by binding 3′-untranslated region (3′-UTR) of target messenger RNA [14]. Recent studies were originally identified miRNAs as crucial regulator of glioma biology [15]. Therefore, if we want to investigate novel methods to safely open the BTB, we need to consider the regulatory roles of those ncRNAs including lncRNAs and miRNAs on BTB permeability.

Taurine upregulated gene 1 (TUG1), an lncRNA whose gene is located at chromosome 22q12, was originally identified that contributes to the forming of photoreceptors and plays crucial roles in retinal development [16, 17]. Beside, TUG1 is required for regulating carcinogenesis in several of tumors, such as osteosarcoma [18] and melanoma [19].

MicroRNA-144 (miR-144) has been originally identified as an erythroid-specific manner, which plays a crucial role in erythroid development [20, 21]. In the field of tumor study, previous studies revealed that miR-144 had significantly dysregulated in tumors, but where it is up-regulated or down-regulated in tumors was not clear based on recent published results. A meta-analysis has suggested that the genomic locus of miR-144 (17q11) was frequently lost in several cancers [22]. In other hand, Zhang et al reported that microRNA-144 (miR-144) was an up-regulated gene in nasopharyngeal carcinoma and acted as an oncogene [23]. In addition, miR-144 also contributed to the regulation of functioning of the endothelium [24]. However, the expression of TUG1 and miR-144 in brain microvascular endothelial cells and its roles in regulating BTB function remain unclear.

In the present study, the major aim was to investigate the expression of lncRNA-TUG1, microRNA-144 and transcription factors HSF2 in glioma microvascular endothelial cells. Meanwhile, the interaction among lncRNA-TUG1, microRNA-144 and transcription factors HSF2 in regulation in the regulation of BTB permeability and the possible mechanism were also revealed.

## RESULTS

### The expressions of TUG1, miR-144 and HSF2 in human glioma vascular endothelial cells and normal brain vascular endothelial cells

**Figure 1:**
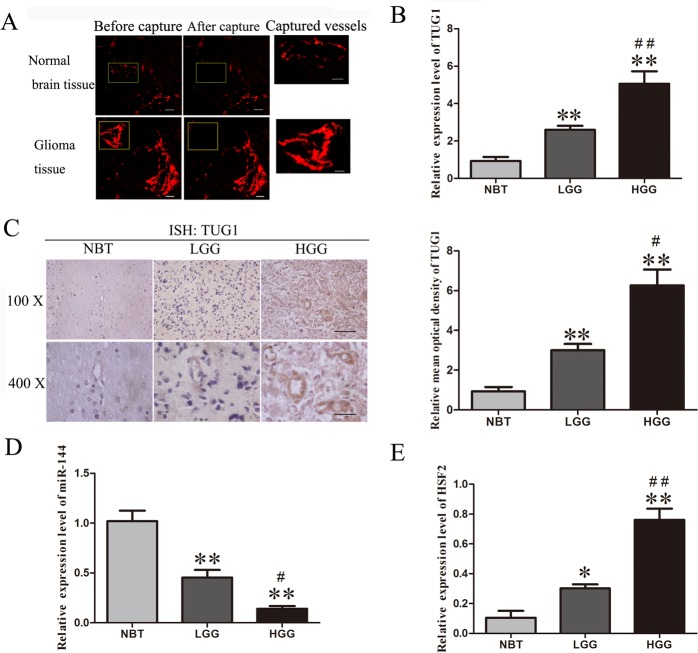
The expressions of TUG1, miR-144 and HSF2 in human glioma vascular endothelial cells and normal brain vascular endothelial cells **A.** Glioma vascular endothelial cells were captured from glioma tissues using laser capture microdissection. LCM capture of UEA-I-stained vessel from glioma or normal brain tissues (lift two panels: magnification, ×400; scale bar, 10μm. Right panel: magnification, ×1000; scale bar, 10μm). **B.** The expression levels of TUG1 in human glioma vascular endothelial cells and normal brain vascular endothelial cells were detected by quantitative real-time PCR. **C.** Representative images of ISH analyses of normal brain tissues and glioma specimens, including low grade glioma (LGG) and high grade glioma (LGG) tissues, were stained by ISH by using a specific Digoxin tag TUG1 probe. The relative mean optical densities of TUG1 staining are shown. The expression levels of miR-144 **D.** and HSF2 **E.** in human glioma vascular endothelial cells and normal brain vascular endothelial cells were detected by quantitative real-time PCR. Data represent mean ± SD (*n* = 5, each). **P < 0.01 vs. normal brain tissues group. ^##^*P* < 0.01 *vs*. low grade glioma tissues group. ^#^*P* < 0.05 *vs*. low grade glioma tissues group.

Glioma vascular endothelial cells were captured from glioma tissues, while normal brain vascular endothelial cells were captured from normal brain tissues (Figure 1A). The expression levels of TUG1, miR-144 and HSF2 in human glioma vascular endothelial cells and normal brain vascular endothelial cells were detected by quantitative real-time PCR (Figure 1B, 1D and 1E).

Quantitative real-time PCR analysis was applied to detect the expression of lncRNA TUG1 in glioma vascular EC captured from low and high grade glioma tissues as well as in EC captured from normal brain (Figure 1B). The expression of TUG1 was significantly increased in low or high-grade glioma group compared with that in the normal brain tissue group (*P* < 0.01). Besides, the expression level of TUG1 in high-grade glioma group was up-regulated compared with that in the low-grade glioma group (*P* < 0.01).

In Situ Hybridizations (ISH) analysis was applied to detect the expression of lncRNA TUG1 in low and high grade glioma tissues as well as in normal brain tissues (Figure 1C). ISH analysis showed that TUG1 was more strongly stained in glioma tissues than normal brain tissues. TUG1 was intensively stained in the cytoplasm of glioma cell, endothelial cell and vessel lumen of glioma tissues. The relative mean optical densities of TUG1 was significantly increased in low or high-grade glioma group compared with that in the normal brain tissue group (*P* < 0.01). Besides, the relative mean optical densities of TUG1 in high-grade glioma group was up-regulated compared with that in the low-grade glioma group (*P* < 0.05).

As showed in Figure 1D, the expression of miR-144 was significantly reduced in low or high-grade glioma group compared with that in the normal brain tissue group (*P* < 0.01). Besides, the expression level of miR-144 in high-grade glioma group was down-regulated compared with that in the low-grade glioma group (*P* < 0.05).

Furthermore, the expression levels of HSF2 were significantly up-regulated in low or high-grade glioma group compared with that in the normal brain tissue group. In addition, the expression levels of HSF2 in high-grade glioma group was higher than that in the low-grade glioma group (*P* < 0.01; Figure 1E).

### The expressions of TUG1, miR-1144 and HSF2 in EC and GEC

**Figure 2:**
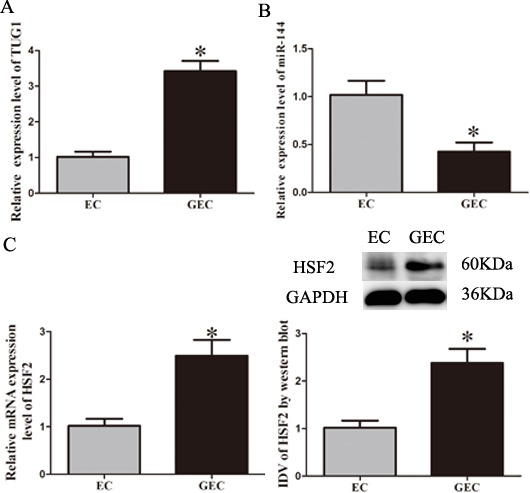
The expressions of TUG1, miR-144 and HSF2 in EC and GEC **A.-B.** Relative expression levels of TUG1 and miR-144 in EC or GEC were detected by quantitative real-time PCR. **C.** Relative mRNA and protein expression of HSF2 in EC or GEC were detected, using GAPDH as an endogenous control. Representative protein expression and their integrated light density values (IDVs) of HSF2 are shown. Data represent mean ± SD (*n* = 5, each). ^*^P < 0.05 *vs*. EC group.

After BTB models were established, quantitative real-time PCR analysis (Figure 2A) showed that the expression of TUG1 in GEC (glioma co-cultured endothelial cells) was significantly up-regulated compared to EC (endothelial cells obtained from BBB model) (*P* < 0.05). The expression of miR-144 in GEC was significantly reduced compared to EC (*P* < 0.05; Figure 2B). In addition, the expression of HSF2 in GEC was significantly up-regulated compared to that in EC (*P* < 0.05; Figure 2C).

### Knockdown of lncRNA TUG1 increased BTB permeability through inhibiting the expression of tight junction related proteins in GEC

**Figure 3:**
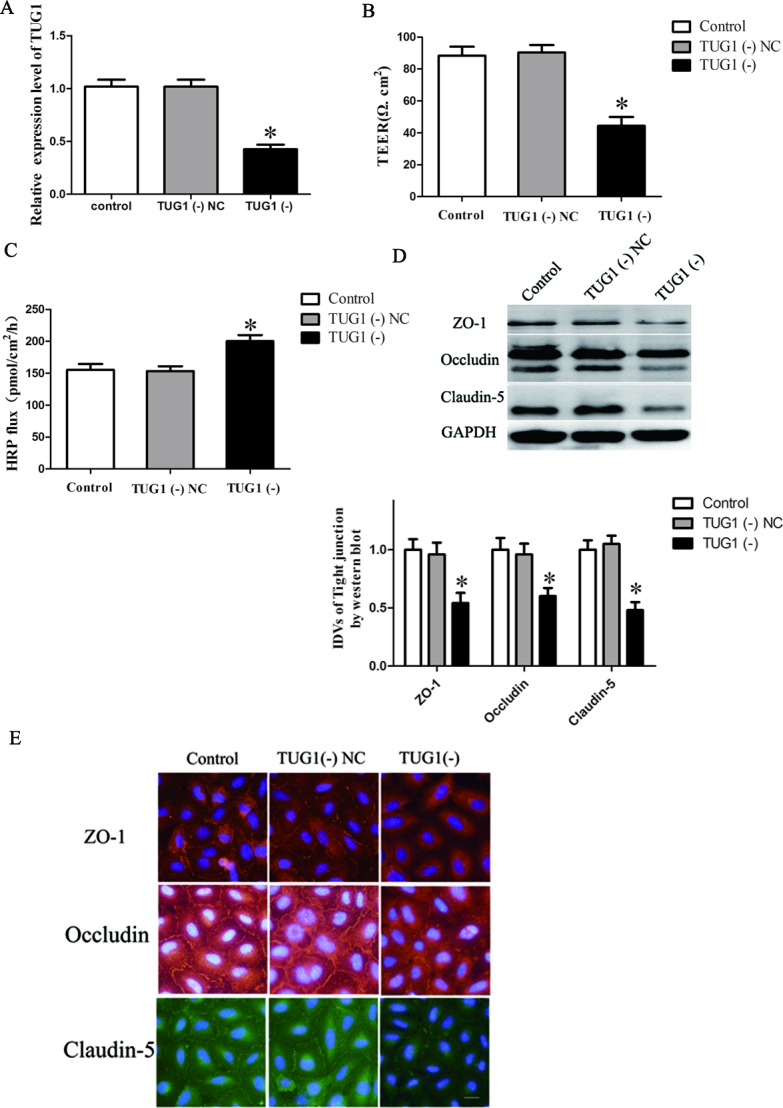
TUG1 regulates BTB permeability and the expression of tight junction related proteins in GEC **A.** *In vitro* BTB models were established using EC with stably knockdown of TUG1, relative expression levels of TUG1 were detected by quantitative real-time PCR. **B.** The Transendothelial electric resistance (TEER) values of BTB were detected after knockdown of TUG1 in GEC. **C.** Permeability assays were performed by HRP flux test. **D.** Western blot analysis of TJ-related proteins ZO-1, occludin, and claudin-5 in GEC. The integrated light density values (IDVs) of protein expression levels of TJ-related proteins are shown. Data represent mean ± SD (*n* = 5, each). *P < 0.05 *vs*. TUG1 (−) NC group. **E.** Immunofluorescence localization of ZO-1, occludin and claudin-5 in GEC. Nuclei are labeled with DAPI. Images are representative of five independent experiments. Original magnification: 100x. Scale bar = 5 um.

*In vitro* BTB models were established using EC with knockdown of lncRNA TUG1. The expression of TUG1 was 65.33% lower in the TUG1 (−) group than that in TUG1 (−) NC group (*P* < 0.05, Figure 3A).

TEER values were detected (Figure 3B). No significant difference in TEER values was found between the control and TUG1 (−) NC groups (*P* > 0.05). TEER values in TUG1 (−) group were significantly lower than that in TUG1 (−) NC group (*P* < 0.05). HRP flux test results were shown in Figure 3C. No significant difference in the penetration rate of HRP was found between the control and TUG1 (−) NC groups (*P* > 0.05); the penetration rate in TUG1 (−) group was higher than that of TUG1 (−) NC group (*P* < 0.05).

The expression of ZO-1, occludin and claudin-5 in EC with knockdown of lncRNA TUG1 was detected by Western blot assay (Figure 3D). Results demonstrated that the expression of tight junction proteins showed no significant difference between control and TUG1 (−) NC groups (*P* > 0.05). However, the expression of these proteins was significantly down-regulated in the TUG1 (−) group compared with the TUG1 (−) NC group (*P* < 0.05).

Immunofluorescence analysis (Figure 3E) revealed that ZO-1, occludin and claudin-5 exhibited a continuous distribution along cell border of the endothelial cells in the control group and were discontinuously distributed at tight junctions in the TUG1 (−) group. In addition, occludin was also expressed in the cytoplasm. Similarly, results of immunofluorescence also confirmed that the expression of occludin, ZO-1 and claudin-5 was significantly decreased in the TUG1 (−) group compared with TUG1 (−) NC group.

### Overexpression of miR-144 increased BTB permeability through inhibiting the expression of tight junction related proteins in GEC

**Figure 4:**
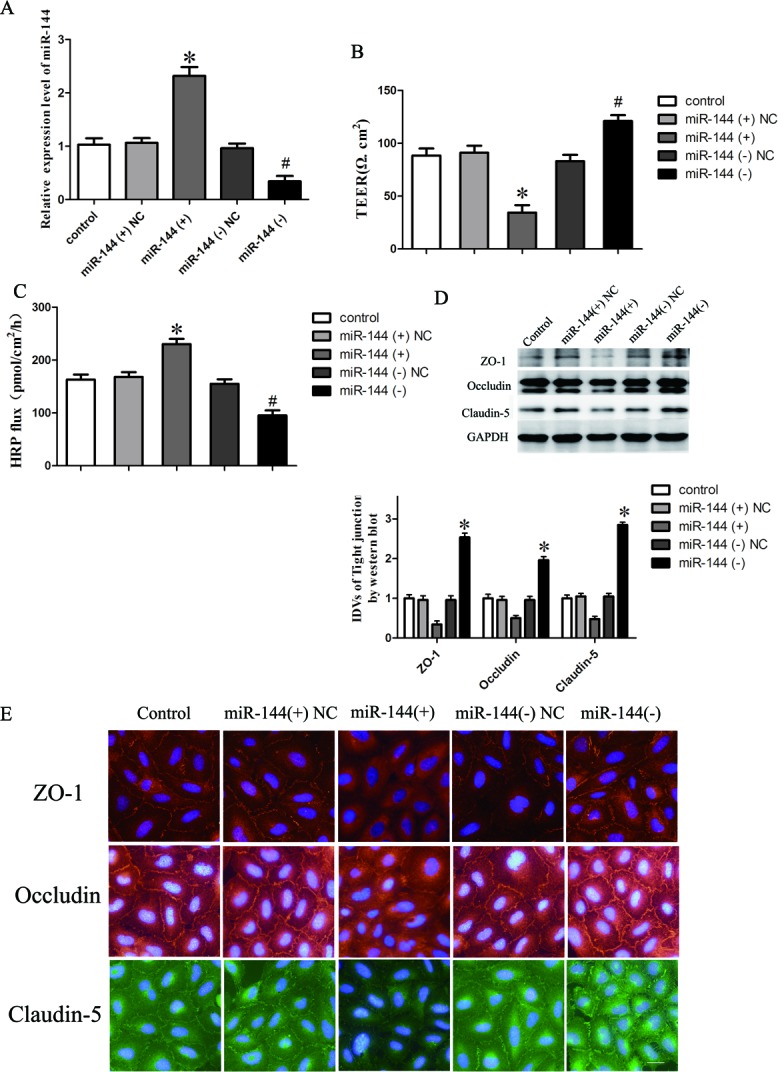
MicroRNA-144 regulates BTB permeability and the expression of tight junction related proteins in GEC **A.** *In vitro* BTB models were established using EC with stably overexpression or knockdown of miR-144, relative expression levels of miR-144 were detected by quantitative real-time PCR. **B.** The Transendothelial electric resistance (TEER) values of BTB were detected after changing the expression of miR-144 in GEC. **C.** Permeability assays were performed by HRP flux test. **D.** Western blot analysis of TJ-related proteins ZO-1, occludin, and claudin-5 in GEC. The integrated light density values (IDVs) of protein expression levels of TJ-related proteins are shown. Data represent mean ± SD (*n* = 5, each). *P < 0.05 *vs*. miR-144 (+) NC. ^#^*P* < 0.05 vs. miR-144 (−) NC. **E.** Immunofluorescence localization of ZO-1, occludin and claudin-5 in GEC. Nuclei are labeled with DAPI. Images are representative of five independent experiments. Original magnification: 100x. Scale bar = 5 um.

We next assessed the functional role of miR-144 in GECs by determining the effects of miRNA overexpression and inhibition on the BTB permeability using TEER and HRP flux test assays, respectively. Overexpression and inhibition of miR-144 were obtained by stable transfection and clones were further generated (Figure 4A). TEER values were detected (Figure 4B). No significant difference in TEER values was found among the control, miR-144 (+) NC and miR-144 (−) NC groups (*P* > 0.05). TEER values in miR-144 (+) group were significantly lower than those in miR-144 (+) NC group (*P* < 0.05), the values in miR-144 (−) group were significantly higher than those in miR-144 (−) NC group (*P* < 0.05).

HRP flux test results were shown in Figure 4C. No significant difference in the penetration rate of HRP was found among the control, miR-144 (+) NC and miR-144 (−) NC groups (*P* > 0.05); the penetration rate in miR-144 (+) group was higher than that of miR-144 (+) NC group (*P* < 0.05) and the level in miR-144 (−) group was lower than that in miR-144 (−) NC group (*P* < 0.05).

The expression of occludin, ZO-1 and claudin-5 in GEC with either stable over-expression or knockdown of miR-144 was detected by western blot assay (Figure 4D). Results demonstrated that the expression of tight junction proteins showed no significant difference among the control, miR-144 (+) NC and miR-144 (−) NC groups (*P* > 0.05). However, the expression of these proteins was significantly down-regulated in the miR-144 (+) group compared with the miR-144 (+) NC group (*P* < 0.05) and was significantly up-regulated in the miR-144 (−) group compared with the miR-144 (−) NC group (*P* < 0.05).

Immunofluorescence analysis (Figure 4E) revealed that ZO-1, occludin and claudin-5 exhibited a continuous distribution along cell border of the endothelial cells in the control group and were discontinuously distributed at tight junctions in the miR-144 (+) group. In addition, occludin was also expressed in the cytoplasm. Similarly, results of immunofluorescence also confirmed that the expression of ZO-1, occludin and claudin-5 was significantly increased in miR-144 (−) group compared with the control group and was reduced in the miR-144 (+) group.

### LncRNA TUG1 was a competing endogenous RNA (ceRNA) of miR-144

**Figure 5:**
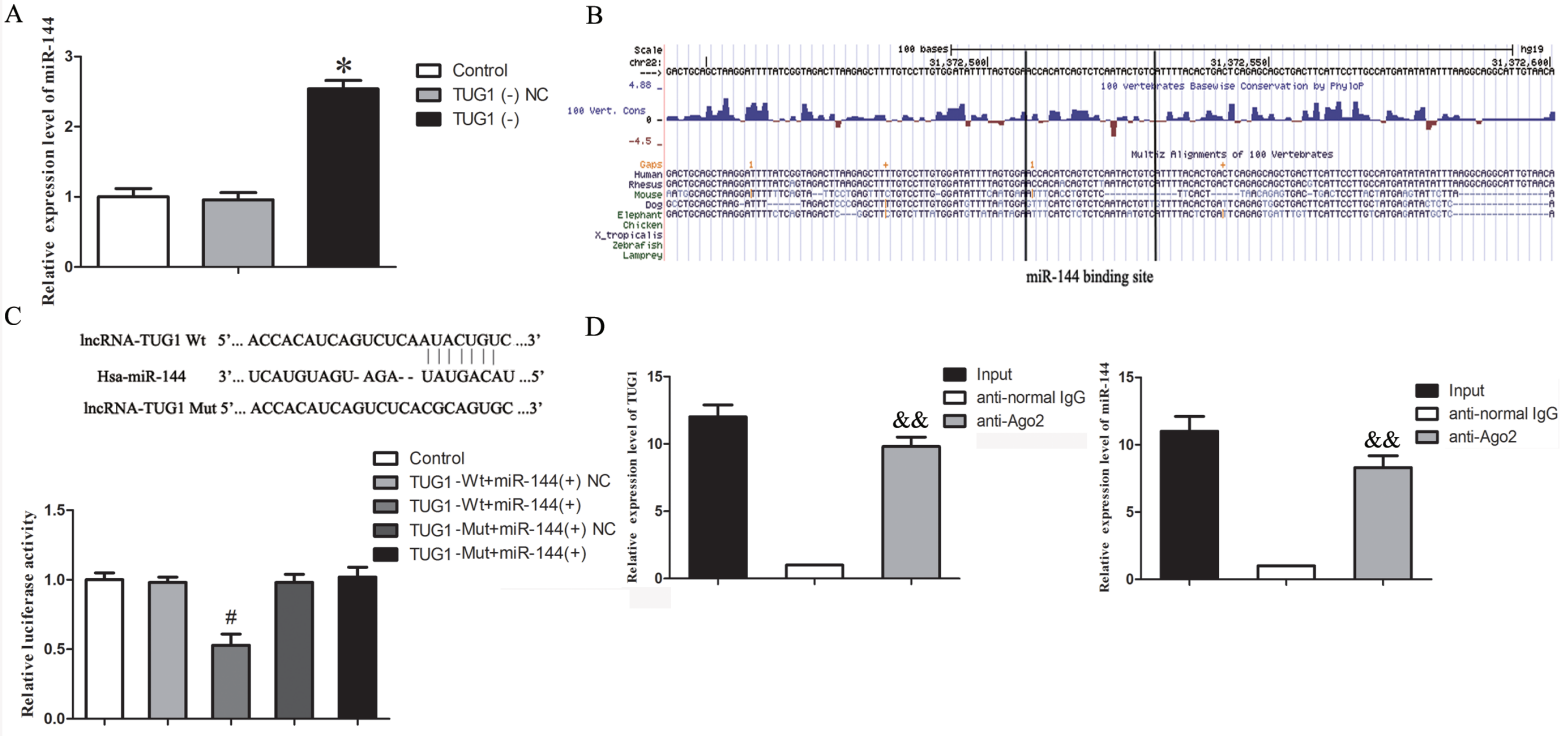
The interaction between LncRNA TUG1 and miR-144 **A.** Effect of knockdown of TUG1 on the expression of miR-144 in GEC. Relative expression levels of miR-144 were detected by quantitative real-time PCR. Data represent mean ± SD (*n* = 5, each). *P < 0.05 *vs*. TUG1 (−) NC group. **B.** Conservation of TUG1 in the binding site of miR-144 was snapshotted from human genome in UCSC Genome Browser. **C.** Relative luciferase activity was performed by dual-luciferase reporter assay. Data represent mean ± SD (*n* = 5, each). ^#^*P* < 0.05 *vs*. TUG1-wt+ miR-144 NC group. **D.** RIP assay were performed using input from cell lysate, normal mouse IgG or anti-Ago2. Relative expression levels of TUG1 and miR-144 were detected by quantitative real-time PCR. Data represent mean ± SD (*n* = 4, each). ^&&^*P* < 0.01 *vs*. anti-normal IgG group.

LncRNA might be a competing endogenous RNA (ceRNA) or a molecular sponge in modulating the expression and biological functions of miRNA [8, 25]. To determine whether miR-144 is regulated by TUG1, bioinformatics analysis (miRanda) of miRNA recognition sequences was performed and the result revealed that the binding sites of miR-144 were presented in TUG1 cDNA. Figure 5A indicated that the stable knockdown of TUG1 significantly increased the expression level of miR-144 (*P* > 0.05). Moreover, the binding site of miR-144 to TUG1 was highly conserved among species (Figure. 5B). To better clarify the underlying mechanism of the lncRNA/miRNA regulatory function, dual-luciferase reporter assay was performed (Figure 5C). The results showed that no significant difference in the relative luciferase activity between TUG1-mut+ miR-144 and TUG1-mut+miR-144 NC groups (*P* > 0.05); co-transfection of pmirGLO-TUG1-wild type and agomir-144 (TUG1-wt+ miR-144 group) greatly reduced the luciferase activity compared with TUG1-wt+ miR-144 NC group (*P* < 0.05).

To determine whether TUG1 is associated with the RNA-induced silencing complex (RISC) complex, we performed RNA-binding protein immunoprecipitation (RIP) assay. RNA level of TUG1 and miR-144 were higher in anti-Ago2 group than that in anti-normal IgG group (*P* < 0.01, Figure. 5D).

### LncRNA TUG1 regulated BTB permeability through inhibiting the expression of tight junction related proteins in GEC by targeting miR-144

**Figure 6:**
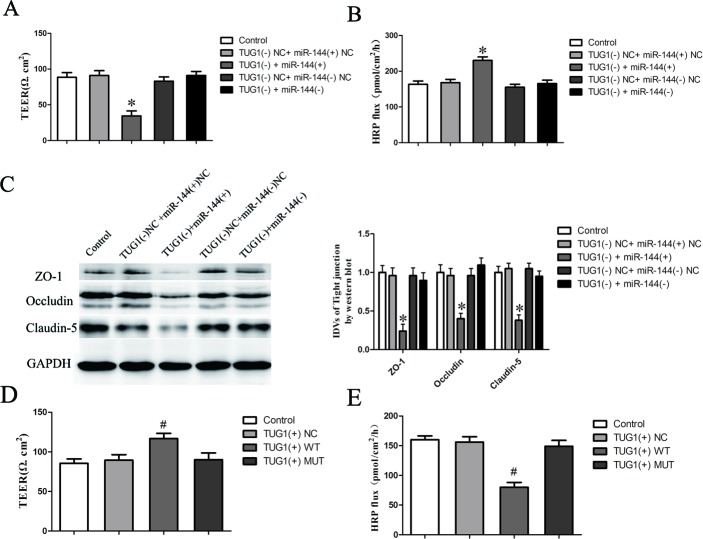
TUG1 regulates BTB permeability through inhibiting the expression of TJ-related proteins in GEC by targeting miR-144 **A.** The Transendothelial electric resistance (TEER) values of BTB were detected after changed miR-144 expression in GEC, which stably knockdown of TUG1 (TUG1 (−)). **B.** Permeability assays were performed by HRP flux test. **C.** Western blot analysis of TJ-related proteins ZO-1, occludin, and claudin-5 in GEC. The integrated light density values (IDVs) of protein expression levels of TJ-related proteins are shown. Data represent mean ± SD (*n* = 5, each). *P < 0.05 *vs*. TUG1+ miR-144 (+) NC group. **D.** The Transendothelial electric resistance (TEER) values of BTB were detected after overexpression of control (TUG1 (+) NC), TUG1 wild type (TUG1 (+) WT) or mutant type (TUG1 (+) MUT) in GEC. **E.** Permeability assays were performed by HRP flux test. Data represent mean ± SD (*n* = 5, each). ^#^*P* < 0.05 *vs*. TUG1 (+) NC group.

Having confirmed that TUG1 was a target of miR-144, the role of miR-144 in lncRNA TUG1-induced regulation of the BTB permeability remains unclear. To clarify whether miR-144 was involved in the TUG1-mediated regulation of the BTB permeability as well as the expression of ZO-1, occludin, and claudin-5 were further investigated. Figure 6A and 6B showed that overexpression of miR-144 in GEC, which stably knockdown of TUG1 (TUG1 (−)), largely reversed the promotion effect of TUG1 (−) on the BTB permeability. Moreover, overexpression of miR-144 largely reversed the expression of ZO-1, occludin, and claudin-5 in GEC down-regulated by TUG1 (−) (Figure 6C). These results strongly suggested that miR-144 played a crucial role in knockdown of TUG1-induced promotion effects on the BTB permeability.

To determine the functional specificity of TUG1 for the miR-144 on the BTB permeability, human full-length TUG1 gene and its mutant of the putative miR-144 binding sequence in TUG1 synthesized and cloned into the lentivirus vector. An empty control lentivirus vectors was used as a control. Cells were then infected with lentivirus or control lentivirus. GFP-positive cells were picked to select TUG1 (+)-NC, TUG1 (+) WT and TUG1 (+)-MUT cells and further propagated.

We next assessed the functional specificity of TUG1 for the miR-144 on the BTB permeability using TEER and HRP flux test assays, respectively. TEER values were detected (Figure 6D). No significant difference in TEER values was found among the control, TUG1 (+)-NC and TUG1 (+)-MUT groups (*P* > 0.05). TEER values in TUG1 (+) WT group were significantly higher than those in TUG1 (+)-NC group (*P* < 0.05).

HRP flux test results were shown in Figure 6E. No significant difference in the penetration rate of HRP was found among the control, TUG1 (+)-NC and TUG1 (+)-MUT groups (*P* > 0.05); the penetration rate in TUG1 (+) WT group was lower than that of TUG1 (+)-NC group (*P* < 0.05).

### MicroRNA-144 inhibited the expression of HSF2 by binding 3′-UTR of HSF2

**Figure 7:**
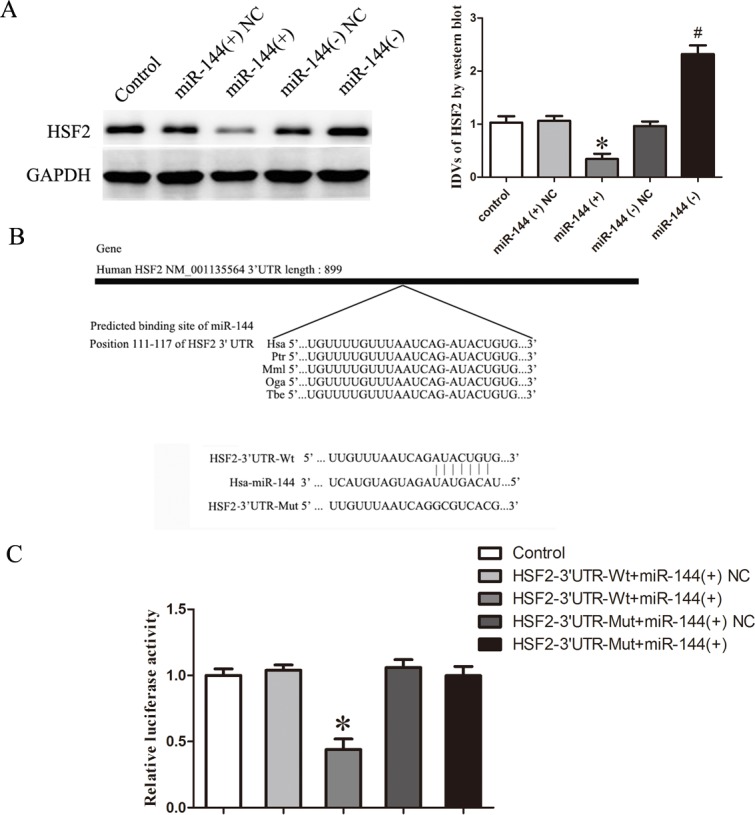
MicroRNA-144 inhibited the expression of HSF2 by binding 3′-UTR of HSF2 **A.** Western blot analysis of HSF2 in GEC after changed the expression of miR-144. **B.** 3′-UTR of HSF2 in the binding site of miR-144 was shown. **C.** Relative luciferase activity was performed by dual-luciferase reporter assay. Data represent mean ± SD (*n* = 5, each). *P < 0.05 *vs*. HSF2-3′UTR-wt+ miR-144 NC group.

To uncover the mRNA targets of miR-144 in GECs, we used bioinformatics databases (http://www.targetscan.org/ and http://pictar.mdc-berlin.de/) to identify potential targets. To experimentally verify these potential targets, cells were transfected with miR-144, protein expression levels of targets were detected by western blotting. It was confirmed that HSF2 was one of the potential targets. Results showed that the protein expression levels of HSF2 were decreased in miR-144 (+) group compared with miR-144 (+) NC group whereas that of miR-144 (−) group was increased compared with miR-144 (−) NC group. These results indicated that miR-144 could inhibit the expression of HSF2 in GECs (Figures 7A).

Moreover, the binding site of miR-144 to HSF2 was highly conserved among species (Figure 7B). To further investigate whether HSF2 was a functional target of miR-144, dual-luciferase reporter assay was performed. The results showed that no significant difference in the relative luciferase activity between HSF2-3′UTR-mut+ miR-144 and HSF2-3′UTR-mut+miR-144 NC groups (*P* > 0.05); the relative luciferase activity of HSF2-3′UTR-wt+miR-144 (+) group was significantly decreased in compared with HSF2-3′UTR-wt + miR-144 (+) NC group (*P* < 0.05; Figure 7C).

### Overexpression of miR-144 increased BTB permeability through inhibiting the expression of tight junction related proteins in GEC by inhibition of HSF2

**Figure 8:**
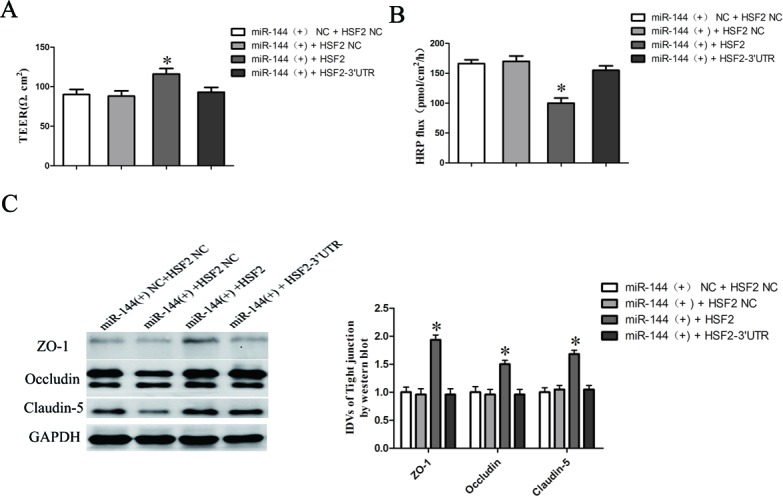
MicroRNA-144 regulates BTB permeability and the expression of tight junction related proteins in GEC by inhibition of HSF2 **A.** The Transendothelial electric resistance (TEER) values of BTB were detected after co-transfected of agomir-144 and HSF with (or without) 3′UTR plasmid in GEC. **C.** Permeability assays were performed by HRP flux test. **D.** Western blot analysis of TJ-related proteins ZO-1, occludin, and claudin-5 in GEC. The integrated light density values (IDVs) of protein expression levels of TJ-related proteins are shown. Data represent mean ± SD (*n* = 5, each). *P < 0.05 *vs*. miR-144 (+) + HSF2 NC group.

Having confirmed that HSF2 was a target gene of miR-144, the role of HSF2 in miR-144-induced regulation of the BTB permeability remains unclear. To clarify whether HSF2 was involved in the miR-144-mediated regulation of the BTB permeability as well as the expression of ZO-1, occludin, and claudin-5 were further investigated. Figure 8A and 8B showed that overexpression of HSF with 3′UTR, which stably overexpression of miR-144, largely reversed the inhibition effect of miR-144 on the BTB permeability. Moreover, overexpression of HSF with 3′UTR largely reversed the expression of ZO-1, occludin, and claudin-5 in GEC down-regulated by miR-144 (Figure 8C).

### HSF2 bound to the promoters of tight junction related proteins in GECs

ChIP assays were performed to clarify whether HSF2 are directly associated with the promoters of ZO-1, occludin and claudin-5 in GECs. The position of transcription start site (TSS) of ZO-1, occludin and claudin-5 was predicted by DBTSS HOME (http://dbtss.hgc.jp/). Analysis of sequences of upstream region (1,500 bp) and downstream region of the TSS using the “TFSEARCH” program (http://mbs.cbrc.jp/reach/db/TFREARCH) indicated the presence of potential HSF2-binding sites.

A putative HSF2 binding sites at −162 positions in ZO-1, a putative HSF2 binding sites at −427 in occludin and one putative HSF2 binding sites at −104 position in claudin-5 were respectively confirmed. Primers were designed to bind sequences flanking the putative HSF2 binding sites. As a negative control, PCR were conducted to amplify in the upstream region of the putative HSF2 binding site that was not expected to associate with HSF2, respectively.

**Figure 9:**
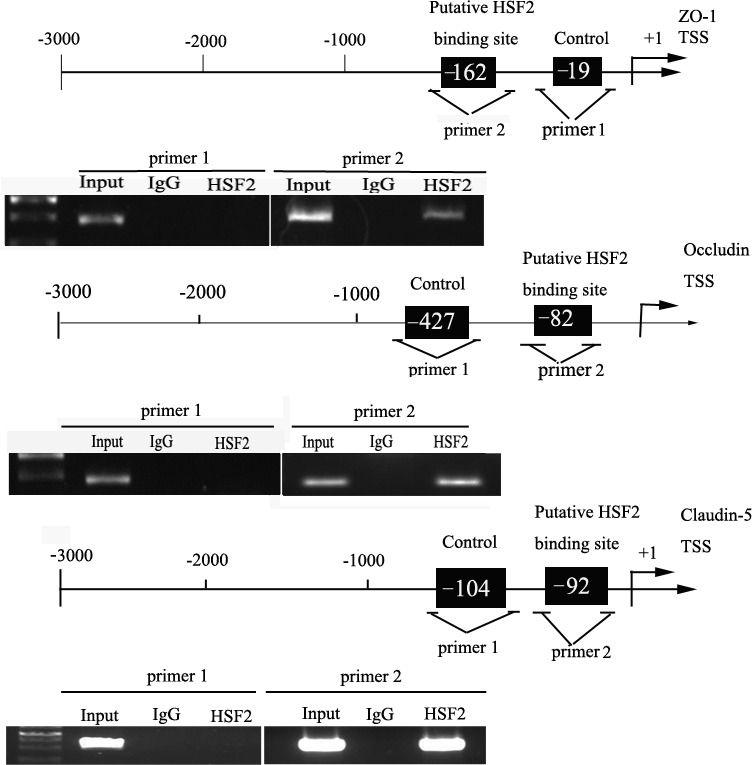
HSF2 bound to the promoters of tight junction related proteins in GECs Schematic representation of the human ZO-1, occludin, and claudin-5 promoter region in 3,000 bp upstream of the transcription start site (TSS, designated as +1). Chromatin immunoprecipitation (ChIP) PCR products for binding sites and an upstream region not expected to associate with HSF2 are amplified by PCR using their specific primers. Image is representative of three independent ChIP experiments.

The results revealed that there was an association of HSF2 with putative binding sites of ZO-1, the putative binding sites of occludin and the putative binding site of claudin-5, but no relationship with all of the negative control groups (Figure 9).

### Overexpression of miR-144 increased *in vivo* BTB permeability of orthotopic xenograft model

To determine the functional role of miR-144 on the BTB permeability *in vivo*, The BALB/c athymic nude mice were received an intracerebral injection of U251 glioma cells into the right striatum. After Eight days later, mice were injected of miR-144 (+) lentivirus or control lentivirus (miR-144 (+) NC) into the tumor-bearing mice brains once via the same needle track where glioma cells were implanted into the mice.

**Figure 10:**
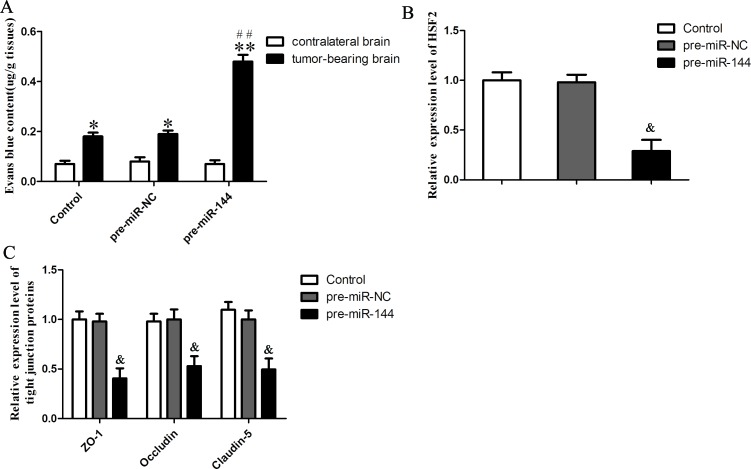
Overexpression of miR-144 increased *in vivo* BTB permeability of orthotopic xenograft model **A.** Content of EB in tumor-bearing brain and contralateral brain after different lentivirus injection 14 days. The expression levels of HSF2 **B.** and tight junction proteins **C.** in in tumor microvessel segments were detected by quantitative real-time PCR. Data represent mean ± SD (*n* = 5, each). *P < 0.05 *vs*. corresponding contralateral brain group. **P < 0.01 *vs*. corresponding contralateral brain group. ^##^*P* < 0.01 *vs*. pre-miR-144 (+) NC tumor-bearing brain group. ^&^*P* < 0.05 *vs*. pre-miR-144 (+) NC group.

The BTB permeability *in vivo* was quantitatively evaluated by extravasation of Evans blue as a marker. Effect on BTB permeability for EB extravasation showed that the brain tumor tissue of orthotopic xenograft model was stained in blue after miR-144 (+) lentivirus injection, while no visible staining was found in contralateral normal brain tissue (Figure 10A). The EB content of tumor-bearing brain significantly increased after Control, miR-144 (+) NC and miR-144 (+) lentivirus injection, while there was no change in the EB content of contralateral brain. Furthermore, the EB content of tumor-bearing brain was significantly higher in miR-144 (+) lentivirus group than in miR-144 (+) NC lentivirus group (*P* < 0.01).

After the animals were euthanized, tumor microvessel segments were isolated as described previously [26, 27]. The expression of HSF2, ZO-1, occludin and claudin-5 in tumor microvessel segments was detected by Real-time PCR assay (Figure 10B, 10C). The results demonstrated that the expression of HSF2 showed no significant difference between control and miR-144 (+) NC groups (*P* > 0.05). However, the expression of HSF2 was significantly down-regulated in the miR-144 (+) group compared with the miR-144 (+) NC group (*P* < 0.05). Besides, the results demonstrated that the expression of tight junction proteins showed no significant difference between control and miR-144 (+) NC groups (*P* > 0.05). However, the expression of these proteins was significantly down-regulated in the miR-144 (+) group compared with the miR-144 (+) NC group (*P* < 0.05).

## DISCUSSION

In the present study, we observed that lncRNA TUG1 was highly expressed in glioma vascular endothelial cells from glioma tissues and glioma co-cultured ECs from *in vitro* BTB model. Knockdown of TUG1 increased BTB permeability, and meanwhile down-regulated the expression of the tight junction proteins ZO-1, occludin, and claudin-5. TUG1 influenced BTB permeability via binding to miR-144, which was down-regulated expressed in GEC. Introduction of miR-144 largely abrogated TUG1-mediated regulation of BTB permeability and the expression of the EC tight junction. Furthermore, Knockdown of TUG1 also down-regulated Heat shock transcription factor 2 (HSF2), a transcription factor of the heat shock transcription factor family, which was defined as a direct and functional downstream target of miR-144. In addition, HSF2 up-regulated the promoter activities and interacted with the promoters of ZO-1, occludin, and claudin-5 in GECs.

Recently, accumulated evidence on lncRNA has indicated that dysregulation of lncRNA may not only affect the biologic processes of tumor, but also modulate the function of vascular endothelial cells [28-30]. Our research provided evidence that the expression of TUG1 was up-regulated in human glioma vascular endothelial cells and glioma co-cultured ECs from *in vitro* BTB model. Taurine Upregulated Gene 1 (TUG1) was first identified as an full-length 6.7 kilobase untranslated RNA molecule (lncRNA TUG1) that lacks any conserved open reading frames, which was expressed in the developing retina, and was necessary for retinal development [16, 17]. TUG1 was accumulates in various tumor tissues such as osteosarcoma [18], esophageal squamous cell carcinoma [31], multiple myeloma [32] and bladder urothelial carcinomas [33], and acted as an oncogenic in tumor growth and development as well. But in another study, knockdown of TUG1 promoted tumor cell proliferation in non-small cell lung carcinoma via regulation of the expression of homeobox B7 (HOXB7) [34]. To explore whether TUG1 might be correlated to the tumorigenesis of glioma, we detected the expression levels of TUG1 in glioma tissues (support Figure 1). The results indicated that TUG1 was significantly up-regulated compared with normal brain tissues. Emerging evidence suggests that TUG1 was expressed in a higher level in various endothelial cells lines including human umbilical vein endothelial cells (HUVEC), cardiac microvasculature (HCMEC-C), lung microvasculature (HMVEC-L), coronary arteries (HCAEC), and aorta (HAEC) [35]. Furthermore, glioma vascular endothelial cells were captured from glioma tissues using laser capture microdissection. We found that the expression of TUG1 in glioma vascular endothelial cells was up-regulated compared with that in normal vascular endothelial cells, which suggested that TUG1 might involve in the regulation of the function of glioma vascular endothelial cells and play a pivotal role in BTB permeability.

Blood-brain barrier consists of tight junctional proteins [36, 37] and serves as a barrier to prevent drug delivery to central nervous system [38]. *In vitro* BTB model is essential for the study on the interaction between endothelial cells and glioma cells. The cell line hCMEC/D3 was traditionally used as an *in vitro* model of the human blood-brain barrier [39, 40] to study various pathological changes in CNS [41] and as well as benefit the development of effective chemotherapy for brain tumors. Results of the present study confirmed that TUG1 was endogenously expressed in hCMEC/D3 cells. Here co-culture of immortalized vascular endothelial hCMEC/D3 and glioma cells according to our previous study [42]. Based on this model, we revealed for the first time that the expression of TUG1 was significantly up-regulated in glioma co-cultured endothelial cells compared with normal astrocytes co-cultured endothelial cells, suggesting that TUG1 might be involved in the regulation of BTB function.

Further, ours data showed that knockdown of TUG1 reduced the TEER values and increased HRP flux, which were all the key indexes to evaluate BTB permeability according to the literature [43, 44]. Above results indicated that TUG1 played a crucial role of TUG1 in regulating BTB permeability. Tight junction proteins, which were composed of cytosolic scaffold proteins ZOs (ZO-1. ZO-2), transmembrane proteins occludin and claudins, junctional adhesion molecule (JAM) et al, were responsible for the structural integrity of BBB and the regulation of BBB permeability [45, 46]. For BTB, when BTB permeability is increased, the expressions of tight junction proteins including ZO-1, occludin, and claudin-5 will be significantly down-regulated [47]. Our data showed that knockdown of TUG1 contributed to increased BTB permeability by down-regulating the expression of ZO-1, occludin, and claudin-5. However, above experiments did not illuminate the potential interactions among TUG1, BTB permeability and tight junction proteins.

Recently, several recent reports have confirmed that a new regulatory mechanism between lncRNAs and miRNAs. LncRNA may function as a competing endogenous RNA (ceRNA) or a molecular sponge in modulating the expression and biological functions of miRNA such as post-transcriptional regulation, suggesting that there might be an inverse correlation between expression of lncRNA and miRNA [8, 12, 25, 48]. To find out whether TUG1 serves as a miRNA sponge, we performed the bioinformatics analysis to explore the potential interactions between them and demonstrated that the expression of miR-144 was significantly promoted in GEC that down-regulated TUG1. Bioinformatics prediction and Dual- luciferase reporter assay verified that miR-144 can bind to TUG1 directly by the putative miRNA response element (MRE). MRE was identified to be a highly conserved sequence by UCSC Genome Browsers, suggesting that MRE might be an important functional sequence element. Moreover, upregulated expression of miR-144 could also suppress the TUG1 expression, whereas down-regulated miR-144 induced a reverse result, which suggested that there was a reciprocal repression between TUG1 and miR-144. To better clarify the underlying mechanism of the lncRNA/miRNA regulatory function, we performed RIP assay. The results provided in the present study support the involvement of RNA-induced silencing complex in this reciprocal repression process, which is consistent with the following literature. LncRNA HOTAIR functions as a competing endogenous RNA to regulate HER2 expression by sponging miR-331-3p in gastric cancer [8]. LncRNA-CHRF acts as an endogenous sponge of miR-489, which down-regulates miR-489 expression levels and regulates the expression of differentiation primary response gene 88 (Myd88) and cardiac hypertrophy [49].

MicroRNA-144 (miR-144), a novel erythroid-specific manner [20, 21], was frequently down-regulated and characterized as a tumor suppressor in various human tumors, such as liposarcomas [50], bladder cancer [51] and follicular thyroid carcinoma [52]. MicroRNA-144 also contributed to the regulation of functioning of the endothelium. MicroRNA-144 contributed to endothelial oxidative stress and impaired endothelial function via binding with isocitrate dehydrogenase 2 (IDH2) [24]. Overexpression of miR-144 resulted in abnormal vascular development of intersegmental vessels by reduced expression of meis1 during zebrafish development [53]. Our results indicated that miR-144 was significantly down-regulated compared with normal brain tissues (support Figure 1). Besides, the expression of miR-144 in GECs was down-regulated compared with that in ECs. Furthermore, overexpression of miR-144 contributed to increased BTB permeability by down-regulating the expression of occludin, ZO-1 and claudin-5, while knockdown of miR-144 had contrary effects. Those suggested that miR-144 might involve in the regulation of the function of GEC and play a pivotal role in BTB permeability.

To verify the hypothesis that TUG1 functions via down-regulating the expression of miR-144, agomiR-144 was used to up-regulate the miR-144 expression in GEC that stably silenced TUG1. The results indicated that overexpression of miR-144 in GEC, which stably knockdown of TUG1, largely reversed the promotion effect of knockdown of TUG1 on the BTB permeability. Moreover, overexpression of miR-144 largely reversed the expression of ZO-1, occludin and claudin-5 in GEC down-regulated by TUG1. Therefore, it may highlight the significance of the interaction between miR-144 and lncRNA-TUG1 in BTB permeability that TUG1-induced promotion effects on the BTB permeability by inhibiting miR-144.

As far as we know, miRNAs negatively regulate target gene expression by binding 3′-UTR of target messenger RNA [14]. Several genes, such as transcript gata2 [54], PTEN [55], mTOR [56] and Meis 1 [57], have been identified as the direct targets of miR-144. Bioinformatics analysis and luciferase assay were indicated that HSF2 was one of the direct targets of miR-144 in regulating the permeability of BTB. HSF2, one of the heat shock transcription (HSF) family, are associated with multiple biologic processes such as heat shock response and spermatogenesis [58, 59]. HSF2 has been reported could be activated by high extracellular potassium and was involved in the up-regulation of alphaB-crystallin gene expression in human glioma U-251MG cells [60]. We observed that HSF2 was highly expressed in glioma endothelial cells (GEC) from glioma tissues, which was consistent with the following literature. Colligin 2 was higher expression in glioma neovasculature, which compared to the normal vasculature of the brain, was associated with overexpression of HSF2 in glioma neovasculature [61]. Having confirmed that HSF2 was a target gene of miR-144, the role of HSF2 in miR-144-induced regulation of the BTB permeability has been clarified. The results showed that overexpression of HSF with 3′UTR, which stably overexpression of miR-144, largely reversed the inhibition effect of miR-144 on the BTB permeability by down-regulating the expression of occludin, ZO-1 and claudin-5.

On the basis of these above findings, ChIP assays were carried out to elucidate whether HSF2 interacted with the promoters of ZO-1, occludin and claudin-5 in GEC. Results showed that HSF2 acted as a transcriptional factor, which bound to the promoter region of tight junction proteins ZO-1, occludin and claudin-5. Several studies showed the transcription of heat shock gene expression, such as hsp70, expression requires the activation and translocation to HSF2 [62, 63]. As a transcriptional factor, HSF2 could activate and translocate several genes. HSF2 was involved in radial neuronal migration though directly regulating the transcription of p35 gene [64]. HSF2 was also found to regulate the transcription of male-specific region of the mouse Y chromosome long arm (MSYq) resident genes during spermatogenesis [65].

In summary, the present study for the first time highlighted the significance of the interaction among lncRNA-TUG1, microRNA-144 and transcription factors HSF2 in regulation of BTB permeability. This interaction can be considered as a potential target for the glioma therapies based on attenuating BTB in the future.

## MATERIALS AND METHODS

### Reagents and antibodies

Endothelial basal medium-2 (EBM-2) was purchased from Lonza (Walkersville, MD, USA). Fetal bovine serum (FBS) “Gold” and 10 mM HEPES were purchased from PAA Laboratories GmbH Laboratories (Pasching, Austria). High-glucose Dulbecco's modified Eagle medium (DMEM), Dulbecco's modified Eagle medium/F12 mixed medium and FBS were purchased from Gibco (Life Technologies, Carlsbad, CA, USA). Penicillin-streptomycin, chemically defined lipid concentrate were purchased from Invitrogen (Life Technologies, Carlsbad, CA, USA). Cultrex rat collagen-I was obtained from R&D Systems (Minneapolis, MN, USA), Human basic fibroblast growth factor (bFGF), hydrocortisone and ascorbic acid were obtained from Sigma-Aldrich (St Louis, MO, USA). Horseradish peroxidase (HRP, NW: 40 KDa) were purchased from Sigma-Aldrich (Sigma-Aldrich, St Louis, MO, USA). EZ-Magna RNA-binding protein immunoprecipitation kit was obtained from Millipore (Millipore, Billerica, MA, USA).

Rabbit polyclonal anti-Occludin (Ab31721) and mouse monoclonal anti-GAPDH (Ab8245) antibodies were obtained from Abcam (Abcam, Cambridge, MA, USA). Rabbit polyclonal anti-ZO-1 (61-7300) and anti-Claudin-5 (34-1600) antibodies were obtained from Zymed Laboratories (Life Technologies, Carlsbad, CA, USA). Rabbit polyclonal anti-HSF2 (sc-13056X) antibody was obtained from Santa Cruz Biotechnology (Dallas, Texas USA). Mouse anti-Argonaute2 (Ago2) antibody and normal mouse IgG were obtained from Millipore (Millipore, Billerica, MA, USA).

### Patients and specimens

Human glioma specimens were obtained from patients diagnosed with glioma who underwent surgery at the Department of Neurosurgery of Shengjing Hospital, China Medical University, from January 2014 to July 2014. The research methods in our study were approved by the Institutional Review Board at Shengjing Hospital of China Medical University. All participants provided their written informed consent and the hospital ethical committee approved the experiments. All specimens were immediately frozen and preserved in liquid nitrogen after surgical resection. Glioma specimens were classified into four grades by two experienced clinical pathologists according to the WHO classification of tumors in the central nervous system (2007): low-grade glioma tissues (WHO I-II, *n* = 5) and high-grade glioma (WHO III-IV, *n* = 5). Specimens of normal brain tissues obtained from fresh autopsy material (donation from individuals who died in traffic accident and confirmed to be free of any prior pathologically detectable conditions) were used as negative control (*n* = 5).

### Laser capture microdissection and Real-time PCR assay

Glioma and normal brain tissue specimens were frozen-sectioned at 10 um thickness using Microtome Cryostat (MICROM International GmbH, Walldorf, Germany). LCM was performed according to previously description [66]. Vessels in glioma (or normal brain) sections were stained by fluorescent dye-tagged lectin, Ulex europaeus lectin I (UEA-I) (Vector Laboratories, Burlington, ON, Canada) to select the ones allowing for clear identification of vessels. Then Immunofluorescence stained slides were placed on ArcturusXT™ LCM instrument (Applied Biosystems, USA). The magnification was adjusted for optimal visualisation. The glioma endothelial cell (GEC) of glioma specimens or normal endothelial cell (EC) of normal brain tissues were respectively captured and transferred onto CapSure^®^ HS LCM Caps (Invitrogen, USA). Parameters were as follows: 50 mV for power, 0.7 ms for duration time and 7.5 μm for laser spot size.

Total RNA was extracted from pooled vessels captured with Trizol reagent, as described by the manufacturer (Invitrogen, Carlsbad, CA, USA). The cDNA was synthesized from total RNA using High Capacity cDNA Reverse Transcription Kits (Applied Biosystems, Foster City, CA, USA). Real-time PCR was performed by TaqMan^®^ Universal Master Mix II (Applied Biosystems, Foster City, CA, USA) by the following gene expression assays (Taqman^®^, Applied Biosystems): the probe for TUG1 (Hs00215501_m1), HSF2(Hs00988308_m1) and GAPDH (Hs03929097_g1). For quantification of miR-144 expression, reverse transcription and real-time PCR amplification were carried out using Taqman MicroRNA Reverse Transcription Kit and Taqman Universal Master Mix II by the following TaqMan MicroRNA Assay (Applied Biosystems, Foster City, CA, USA): the probe for of miR-144(002676) and endogenous control U6 (001973).

### *In situ* hybridizations (ISH) assay

In situ hybridization staining were performed on fresh paraffin sections (4 um). Section in situ hybridizations were performed using Human TUG1 ISH Detection Kit (BOSTER, Wuhan, China) as described by the manufacturer. In brief, Paraffin embedded slides were dewaxed, rehydrated and incubated in 0.3% H_2_O_2_ for ten minutes to inhibit endogenous peroxidase activity before treated by trypsin. The specific 5`-DIG labeled probes against human TUG1 (Sequence 1: TCCTATTTAAATAAGCCTATTTTATCCTTTGGCCC; Sequence 2: TAATCGAAAGTTAACATTGTCTGAAAAGTTTTGTT; Sequence 3: GGGATATGTGAGCTGTTTCTATGCATAATGGATAT) were used for in situ analysis. Subsequently, slides were incubating overnight at 4°C with hybridization probe. Slides were washed in phosphate-buffered saline (PBS), and then were blocking with 10% normal goat serum for 10 minutes. Subsequently, slides were incubating one hour at 37°C with Rabbit anti-DIG primary antibody, and then incubated with biotinylated goat anti-rabbit IgG (anti-mouse IgG, BOSTER, Wuhan, China) for one hour at 37°C. After incubation with avidin-biotin-peroxidase complex for 10 minutes, samples were stained with three, 3-diaminobenzidine. A Scramble 5`-DIG labeled probe (Sequence: GTGTAACACGTCTATACGCCCA, # 300514-01, EXIQON, Danmark) was used as the negative control. The beta-actin 5`-DIG labeled probe (Sequence: GTGTAACACGTCTATACGCCCA, # 300514-01, EXIQON, Danmark) was used as the positive control. The stainings was analyzed by Olympus DP71immunofluorescence microscopy (Olympus, Tokyo, Japan) and merged with Chemi Imager 5500 V2.03 software.

### Cell culture

The human cerebral microvascular endothelial cell line hCMEC/D3 was provided by Dr. Couraud (Institut Cochin, Paris, France). The cells (passage 30–35) were cultured in culture flasks coated with 150 μg/ml of cultrex rat collagen I. The culture medium contained endothelial basal medium (EBM-2) supplemented with 5% FBS “Gold”, 1% penicillin-streptomycin, 1% chemically defined lipid concentrate, 1 ng/ml bFGF, 1.4 μM hydrocortisone, 5 μg/ml ascorbic acid and 10 mM HEPES. Cells were maintained in a humidified incubator at 37 °C and 5% CO_2_ and medium was refreshed every 48 hours.

Normal human astrocytes (NHA, passage 5–12) and human brain vascular pericytes (HBVP, passage 7–13) were purchased from the Sciencell Research Laboratories (Carlsbad, CA, USA). The culture medium for NHA and HBVP cells was prepared according to the instruction of the manufacturer. Human glioma cell lines U251 MG, U87MG, U118MG and human embryonic kidney 293T cells were obtained from the Shanghai Institutes for Biological Sciences Cell Resource Center (Shanghai, China). U251MG cells were cultured in DMEM/F12 medium supplemented with 10% FBS. Human glioma cell U87MG, U118MG and embryonic kidney 293T cells were cultured in high glucose DMEM medium supplemented with 10% FBS. All cells were maintained in a humidified incubator at 37 °C with 5% CO_2_.

Primary glioma cells were isolated from intraoperative primary glioma samples from patients. Detailed culture methodology has been previously described [67, 68]. Specimens were obtained freshly from the operating room following protocols approved by the institutional review board of Shengjing Hospital of China Medical University and each patient signed a consent form to participate this study. Primary glioma cells were subsequently cultured in DMEM/F12 medium supplemented with 10% FBS at 37 °C with 5% CO_2_.

### Establishment of *in vitro* blood-tumor barrier (or BBB) models

*In vitro* co-culture BBB and BTB models were established as our previously described [42]. Human brain vascular pericytes were seeded (2×10^4^ cells/cm^2^) on the lower sides of filter membranes of Transwell inserts filters (both sides pre-coated with 150 μg/ml of Cultrex Rat Collagen I; 0.4 μm pore size; Corning, NY, USA). Pericytes cells were allowed to adhere overnight, and then hCMEC/D3 cells (2×10^5^ cells/cm^2^) were seeded into the upper chambers of Transwell inserts in 6-well plate.

For the BBB models, normal human astrocytes were seeded at the density of 2× 10^5^ cells/cm^2^ onto the 6-well culture plate and incubation thereafter was 24 hours (37°C, 5% CO_2_).

For the BTB models, human glioma cells were seeded at the same density onto the 6-well culture plate and incubation thereafter was 24 hours (37°C, 5% CO_2_).

The inserts placed in the well of the 6-well culture plates containing Astrocytes (for BBB) or glioma cells (for BTB). The medium was then renewed every 48 hours. After co-culturing 96 hours, normal control endothelial cells (EC) were the endothelial cells obtained from *in vitro* BBB models; glioma co-cultured endothelial cells (GEC) were the endothelial cell from BTB model.

### Cell transfection and generation of stable endothelial cell lines

The short hairpin RNA (shRNA) against human TUG1 gene (NR_110492) was constructed in U6/Neo plasmid vector (GenePharma, Shanghai, China). A plasmid carrying a non-targeting control sequence was used as a transfection control.

Oligonucleotides encoding miR-144 precursor and anti-miR-144 precursor were ligated into the pGPH1/Neo (miR-144 (+)) and pGPU6/Neo (miR-144 (−)) plasmid vectors (GenePharma, Shanghai, China), respectively. Human full-length HSF2 gene (NM_004506.2) with (or without) its 3′-UTR sequences were ligated into pGCMV/MCS/Neo vector (GenePharma, Shanghai, China). The empty vector was used as a negative control. Short hairpin RNA (shRNA) against HSF2 gene was constructed in pGCMV/MCS/Neo plasmid vector (GenePharma, Shanghai, China). A plasmid carrying a non-targeting control sequence was used as a transfection control.

Endothelial cells at approximately 60%–80% confluence were transfected using Opti-MEM I and Lipofectamine LTX Reagent (Life Technologies Corporation, Carlsbad, CA, USA) according to the manufacturer's instructions. The stably transfected cells were selected by the culture medium containing 0.4 mg/ml Geneticin (G418; Invitrogen, CA, USA). After approximately four weeks, G418-resistant cell clones were established. Stable cell lines transfected efficiencies were assessed by real-time PCR.

Furthermore, the miR-144 agonist (agomir-144), negative control of agomir (agomir-NC), miR-144 antagonist (antagomir-144), and negative control of antagomir (antagomir-NC; GenePharma, Shanghai, China) were transiently transfected into endothelial cell which stably knockdown of TUG1 according to the manufacturer's instructions, respectively, according to protocols of Lipofectamine 2000 Reagents (Life Technologies Corporation, Carlsbad, CA, USA).

### Transendothelial electric resistance (TEER) measurements and permeability measurement

To measure the integrity of the BTB, transendothelial electrical resistance assay was performed with millicell-ERS apparatus (Millipore, Billerica, MA, USA) after *in vitro* BTB models were successfully established. In each measurement, TEER was recorded 30 minutes after the medium exchange at room temperature to ensure temperature equilibration and uniformity of culture environment. Collagen coated Transwell inserts without cells were used to correct for background resistance. The final resistance (Ω·cm^2^) was calculated by subtracting background resistance from measured barrier resistance, and then multiplied by the effective surface area of the filter membrane.

After BTB models were established, 0.1M PBS containing 0.5 μM HRP was added into the upper compartment of the Transwell system. One hour later, the medium in the lower chamber was collected and the HRP content of the samples was assayed colorimetrically. The HRP flux was expressed as pmol passed per cm^2^ surface area per hour.

### Western blot assay and immunofluorescence assays

Cell total protein was extracted in RIPA buffer (Beyotime Institute of Biotechnology, Jiangsu, China) supplemented with protease inhibitors (10 mg/ml aprotinin, 10 mg/ml phenyl-methylsulfonyl chloride (PMSF) and 50 mM sodium orthovanadate) and centrifuged at 14000 × g for 10 minutes at 4°C. The protein concentration of the supernatant was determined with the BCA protein assay kit (Beyotime Institute of Biotechnology, Jiangsu, China). Total cell lysates containing 40 μg of protein were fractionated using SDS-PAGE and transferred onto polyvinylidene fluoride (PVDF) membranes (Millipore, USA). After blocking with 5% non-fat dry milk in TBST for 2 hours, membranes were incubated with primary antibodies (anti-GAPDH antibody diluted at 1:1000; anti-HSF2, anti-ZO-1 antibody diluted at 1:500; anti-occludin, anti-claudin-5, antibody diluted at 1:250) at 4°C overnight. After three washes with PBS-Tween (20mM Tris, 137mM NaCl, 0.1% Tween-20, pH 7.6) membranes were incubated with the corresponding HRP-conjugated secondary antibody diluted at 1:5000 at room temperature for 2 hours. Protein bands were visualized by ECL (Santa Cruz Biotechnology, USA) and detected using the ECL Detection System (Thermo Scientific, USA). Then the Protein bands were scanned using Chemi Imager 5500 V2.03 software, and integrated light density values (IDVs) were calculated by Fluor Chen 2.0 software and normalized with those of GAPDH.

Endothelial cells on insert filters were fixed with 4% paraformaldehyde for 20 minutes and blocked with 5% BSA in PBS for 2 hours at room temperature. Cells were incubated with primary antibodies (anti-ZO-1, anti-occludin, anti-claudin-5 antibodies diluted at 1:50) at 4°C overnight. After three washes with PBS, cells were incubated with fluorophore-conjugated secondary antibodies for 2 hours. DAPI (4′, 6′-diamidino-2-phenylindole) was applied to the samples after the final wash to visualize cell nuclei. The stainings was analyzed by Olympus DP71immunofluorescence microscopy (Olympus, Tokyo, Japan) and merged with Chemi Imager 5500 V2.03 software.

### Bioinformatics prediction and dual- luciferase reporter assays

The potential miR-144 binding sites of TUG1 predicted by computer-aided algorithms were obtained from microRNA.org-target program (www.microRNA.org). The putative miR-144 target binding sequence in TUG1 and its mutant of the binding sites were synthesized and cloned downstream of the luciferase gene in the pmirGLO luciferase vector (Promega, Madison, WI, USA).

The target genes of miR-144 were predicted with the help of computer-aided algorithms: TargetScan (http://www.targetscan.org). To examine whether miR-144 targets HSF2 directly, we constructed wild-type HSF2-3′UTR reporter plasmid (HSF2-wt) and mutated-type HSF2-3′UTR reporter plasmid (HSF2-Mut) with pmirGLO-promoter vector.

HEK-293 cells were seeded in 96-well plates for 24 hours, and cells at 60%-80% confluence were co-transfected with wild-type or mutated pmirGLO-TUG1 (pmirGLO-HSF2) reporter plasmid and agomir-144 or agomir-NC. The luciferase activity was measured 48 hours after transfection using the Dual-Luciferase Reporter Assay System (Promega, Madison, WI USA). The relative luciferase activity was expressed as the ratio of firefly luciferase activity to renilla luciferase activity.

### RNA-binding protein immunoprecipitation (RIP) assay

RNA-binding protein immunoprecipitation (RIP) was assayed using a Magna RNA-binding protein immunoprecipitation kit (Millipore, Billerica, MA, USA) according to the instruction provided by the manufacturer. Briefly, ECs were lysed in complete RNA lysis buffer. whole cell lysate was incubated with RIP buffer containing magnetic beads conjugated with human anti-Argonaute2 (Ago2) antibody (Millipore, Billerica, MA, USA), and negative control normal mouse IgG (Millipore, Billerica, MA, USA). Samples were incubated with Proteinase K and then immunoprecipitated RNA was isolated. The RNA concentration was measured by a spectrophotometer (NanoDrop, Thermo Scientific, MA, USA) and the RNA quality assessed using a bioanalyser (Agilent, Santa Clara, CA, USA). Furthermore, purified RNAs extracted and analyzed by qRT-PCR to demonstrate the presence of the binding targets.

### Chromatin immunoprecipitation (CHIP) assay

CHIP Assay was performed with Simple ChIP Enzymatic Chromatin IP Kit (Cell signaling Technology, Danvers, Massachusetts, USA) according to the manufacturer's instruction. In brief, cells were crosslinked with 1% formaldehyde in culture medium for 10 minutes and treated with glycine for 5 minutes at room temperature. These cells were collected in lysis buffer containing PMSF. Micrococcal Nuclease was used to digest chromatin and incubate for 20 minutes at 37°C with frequent mixing. Immunoprecipitation was incubated with 3 ug of anti-HSF2 antibody (Santa Cruz Biotechnology, CA, USA) followed by immunoprecipitation with Protein G Agarose Beads in each sample during an overnight incubation at 4 °C with gentle shaking. Normal rabbit IgG was used as a negative control. While 2% input reference was removed and stored at −20°C before antibody supplemental. The DNA crosslinks was reversed by 5mol/L NaCl and Proteinase K at 65°C for two hours and then DNA was purified. Immunoprecipitated DNA was amplified by PCR using their specific primers (as Supplementary Table 1). In each PCR reaction, the corresponding inputs were taken in parallel for PCR validation. Image is representative of three independent ChIP experiments.

### Lentivirus vector construction and infection

Human full-length TUG1 gene and its mutant of the putative miR-144 binding sequence in TUG1 synthesized and cloned into the LV3-CMV-GFP-Puro vector (GenePharma, Shanghai, China), respectively. An empty control lentivirus vectors was used as a control. Lentiviral vectors and packaging vectors were cotransfected into HEK 293T cells using Lipofectamine 3000 (Life Technologies Corporation, Carlsbad, CA, USA). Virus particles were harvested 48 h after transfection. Cells were then infected with lentivirus or control lentivirus. GFP-positive cells were picked to select TUG1 (+)-NC, TUG1 (+) WT and TUG1 (+)-MUT cells and further propagated.

### Orthotopic xenograft model

The male BALB/c athymic nude mice (*n* = 5 per group, 4-6 weeks old) were purchased from Cancer Institute of the Chinese Academy of Medical Science. All experimental animal procedures were conducted strictly in accordance with the Guide for the Care and Use of Laboratory Animals, and approved by the Animal Care and Use Committee of the Shengjing Hospital.

Human glioma U251 cells were cultured in DMEM/F12 medium supplemented with 10% FBS at 37 °C and 5% CO_2_, and harvested at log phase by centrifugation. The mice were anesthetized with chloral hydrate (350 mg/kg), and then received an intracerebral injection of 1×10^6^ U251 glioma cells into the right striatum (3 mm lateral, 1 mm anterior to the bregma, 4-mm depth from the skull surface) using a Hamilton syringe (Hamilton Company, Reno, NV, USA) and stereotaxic apparatus. After Eight days later, mice were injected of miR-144 (+) lentivirus or control lentivirus (miR-144(+) NC) into the tumor-bearing mice brains once at a dose of 0.5 billion plaque forming units (PFU) via the same needle track where glioma cells were implanted into the mice. Human pre-miR-144 was synthesized and cloned into the pLenti6.3/V5-DEST (GenePharma, Shanghai, China). An empty control lentivirus vectors was used as a control. Data were collected from 5 animals per condition.

### Isolation tumor microvessel segments and Real-time PCR assay

After the animals were euthanized, tumor microvessel segments were isolated as followed the procedures previously reported [26, 27]. The tissue preparation procedure was conducted at 4 °C. The whole tumor was dissected and placed in cold D-Hanks' solution (without Ca^2+^ and Mg^2+^), manually minced with eye scissors, and subsequently homogenized in a glass homogenizer. The tissues were washed with cold D-Hanks' solution and centrifuged. The sediment was re-suspended in cold DMEM containing 15% dextran (Sigma-Aldrich) and re-centrifugated at 10,000×g for 15 minutes to remove myelin, which was concentrated in the upper layer of the supernatant. The remaining sediment was washed twice in DMEM and centrifugated at 3000×g for 5 minutes. The ultimate pellet of tumor microvessel segments was collected, snap frozen, and stored at −80 °C.

Total RNA was extracted from tumor microvessel segments with Trizol reagent, as described previously. The cDNA was synthesized from total RNA using High Capacity cDNA Reverse Transcription Kits (Applied Biosystems, Foster City, CA, USA). Real-time PCR was performed by TaqMan^®^ Universal Master Mix II (Applied Biosystems, Foster City, CA, USA) by the following gene expression assays (Taqman^®^, Applied Biosystems): the probe for HSF2 (Mm00434027_m1), Occludin (Mm01340464_g1), ZO-1 (Mm00493699_m1), claudin-5 (Mm00727012_s1) and GAPDH (Mm99999915_g1). For quantification of miR-144 expression, reverse transcription and real-time PCR amplification were carried out using Taqman MicroRNA Reverse Transcription Kit and Taqman Universal Master Mix II by the following TaqMan MicroRNA Assay (Applied Biosystems, Foster City, CA, USA): the probe for of miR-144(002676) and endogenous control U6 (001973).

### Measurement of BTB permeability by Evans blue (EB) *in vivo*

The BTB permeability was quantitatively evaluated by extravasation of EB as a marker [26, 69]. Briefly, 2% EB in saline (2 mg/kg) was injected intravenously for 2 hours before ventricular perfusion. Mice were injected with miR-144 (+) lentivirus or control lentivirus into the tumor-bearing mice brains once via the same needle track. 14 days later, all the mice were deeply anesthetized with 10% chloral hydrate and perfused with heparinized saline through the cardiac ventricle until colorless perfusion fluid was obtained from the atrium. After the animals were euthanized and the brains were removed, the hemispheres of brain were separated along the interhemispheric plane. Both hemispheres were weighed and immersed into formamide (1 ml/100 mg) at 60 °C for 24 hours. The supernatant was obtained, and its optical density was determined with a spectrophotometer (Shimadzu, Japan) at 620 nm. The concentration of dye extracted from each brain was compared to standard graph created through the recording of optical densities from serial dilutions of EB in 0.9% sodium chloride solution.

### Statistical analysis

Statistical analysis was performed using SPSS 18.0 statistical software. Data was described as mean ± standard deviation (SD). Student's t-test or One-way ANOVA followed by Bonferroni post-test was used to analyze the difference between two groups. *P* < 0.05 was considered to be statistically significant.

## SUPPLEMENTARY FIGURES AND TABLE


