# Supplementary material for: The long noncoding RNA TUG1 regulates blood-tumor barrier permeability by targeting miR-144
Source: Oncotarget. 2015 Jun 2;6(23):19759–79. doi: 10.18632/oncotarget.4331 (PMC4637319; doi:10.18632/oncotarget.4331)
Supplement: Supplementary file 1 [file oncotarget-06-19759-s001.pdf]

The long noncoding RNA TUG1 regulates blood-tumor barrier permeability by targeting miR-144

Supplementary Material

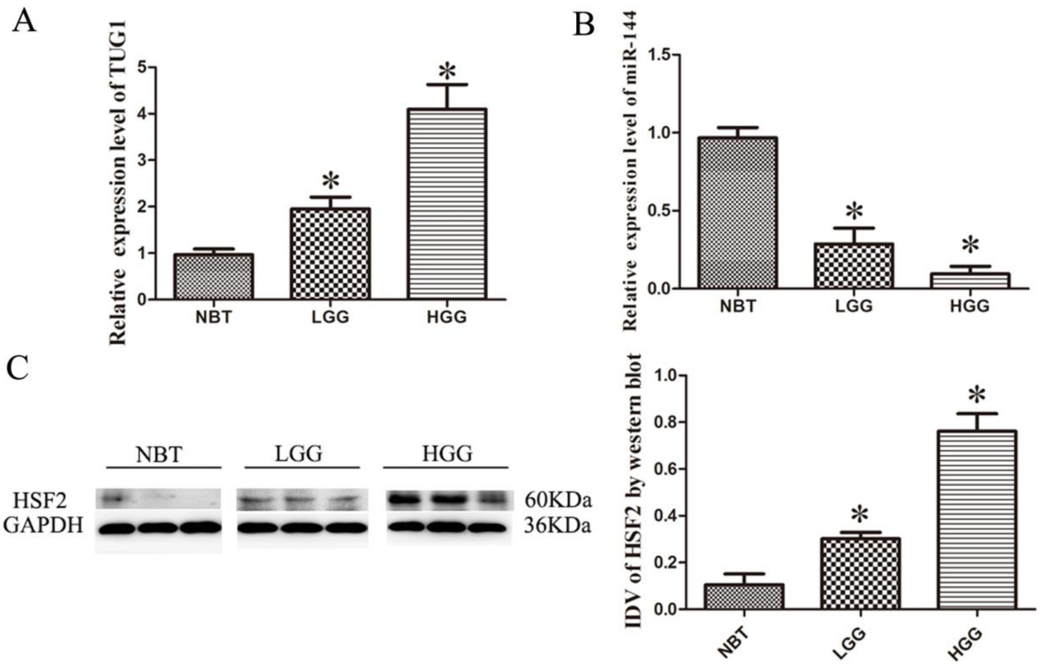

Support Fig. 1: The expressions of TUG1, miR-144 and HSF2 in human glioma tissues and normal brain tissues. The expression levels of TUG1 (A), miR-144 (B) and HSF2 (C) in human glioma tissues and normal brain tissues were detected by quantitative real-time PCR. Data represent mean ± SD (n=5, each). **P*<0.05 vs. normal brain tissues group. (C) Western blot analysis of HSF2 in human glioma tissues and normal brain tissues, using GAPDH as an endogenous control. Representative protein expression and their integrated light density values (IDVs) of HSF2 are shown. Image is representative of three independent experiments.

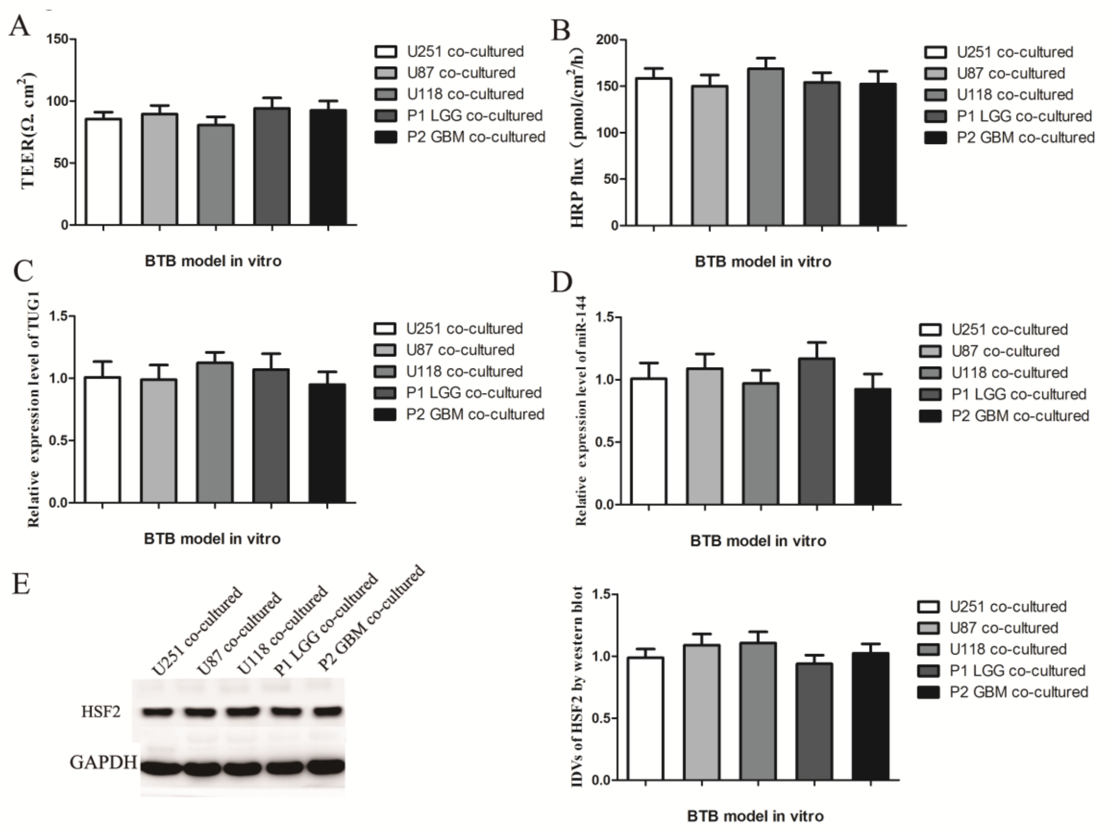

Support Fig. 2 *In vitro* BTB models co-cultured with different glioma cells. (A) The Transendothelial electric resistance (TEER) values of *in vitro* BTB models co-cultured with different glioma cells were detected. (B) Permeability assays were performed by HRP flux test. The expression levels of TUG1 (C) and miR-144 (D) in GEC from BTB models co-cultured with different glioma cells were detected by quantitative real-time PCR. (E) The expression levels of HSF2 in GEC from BTB models co-cultured with different glioma cells were detected by Western blot. Data represent mean ± SD (n=5, each). U251 (U87 or U118) co-cultured: *in vitro* BTB models co-cultured with U251 (U87 or U118) glioma cell; P1 LGG co-cultured: in vitro BTB models co-cultured with 1# patient with low grade glioma; P2 GBM co-cultured: *in vitro* BTB models co-cultured with 2# patient with glioblastoma multiforme.

Supplementary Table 1: Primers used for ChIP assay

Gene	Binding Site or Control	Sequence	Amplicon Size	Annealing Temperature
ZO-1	Primer 1	(F)5’-AATGGTATGGCATAGGAGTGG-3’	165bp	55℃
		(R)5’-TTACGCTTGACAAAGAGGAAG-3’		
	Primer 2	(F)5’-GTGCTGGGATTACAGGCGTGAG-3’	166bp	60℃
		(R)5’-CCTTCTGCAAACCAAACCCTTATT-3’		
Occludin	Primer 1	(F)5’-TTGAGTTTAGGTTTAGGGTGGG-3’	188bp	54℃
		(R)5’-TGGGTTGTTTCTCCTTTGG-3’		
	Primer 2	(F)5’-AGTTCGCTTTCAATGCAGAT-3’	180bp	52℃
		(R)5’-CGGGTGCTGTATTCTATTTCT-3’		
Claudin-5	Primer 1	(F)5’-TGGATGGCACTCAGGAAAG-3’	209bp	53℃
		(R)5’-TTGCGGGCATTGTTGTT-3’		
	Primer 2	(F)5’-ACACCAGTGGACCTTTCG-3’	123bp	51℃
		(R)5’-GCCAACTTGGAGTTTCCTG-3’		
